# The New Impact Factor of International Brazilian Journal of Urology Is 4.3

**DOI:** 10.1590/S1677-5538.IBJU.2026.05.01

**Published:** 2026-08-17

**Authors:** Luciano A. Favorito

**Affiliations:** 1 Universidade do Estado do Rio de Janeiro Unidade de Pesquisa Urogenital Rio de Janeiro RJ Brasil Unidade de Pesquisa Urogenital - Universidade do Estado do Rio de Janeiro - Uerj, Rio de Janeiro, RJ, Brasil; 2 Hospital Federal da Lagoa Serviço de Urologia Rio de Janeiro RJ Brasil Serviço de Urologia, Hospital Federal da Lagoa, Rio de Janeiro, RJ, Brasil

In June 2026 Clarivate released the impact factor of 2026 (Figure-1). To our great satisfaction, The International Brazilian Journal of Urology achieved an impact factor of 4.3 (the cover in this edition). With this impact factor, The International Brazilian Journal of Urology establishes itself as one of the ten most important in the field of urology: a feat that would have been unthinkable in the past. In the present evaluation The International Brazilian Journal of Urology maintained your category ranking (Figure-2). This achievement was made possible only thanks to the Board of the Brazilian Society of Urology, the tireless work of the associate editors, and the prompt availability of the reviewers. Our journal is fully open-access—a unique service made possible by the pioneering spirit of the Brazilian Society of Urology. The Editor-in-Chief extends sincere thanks to everyone involved, especially the technical editorial team, who do outstanding work. We will discuss some articles from this edition below.

**Figure 1:**
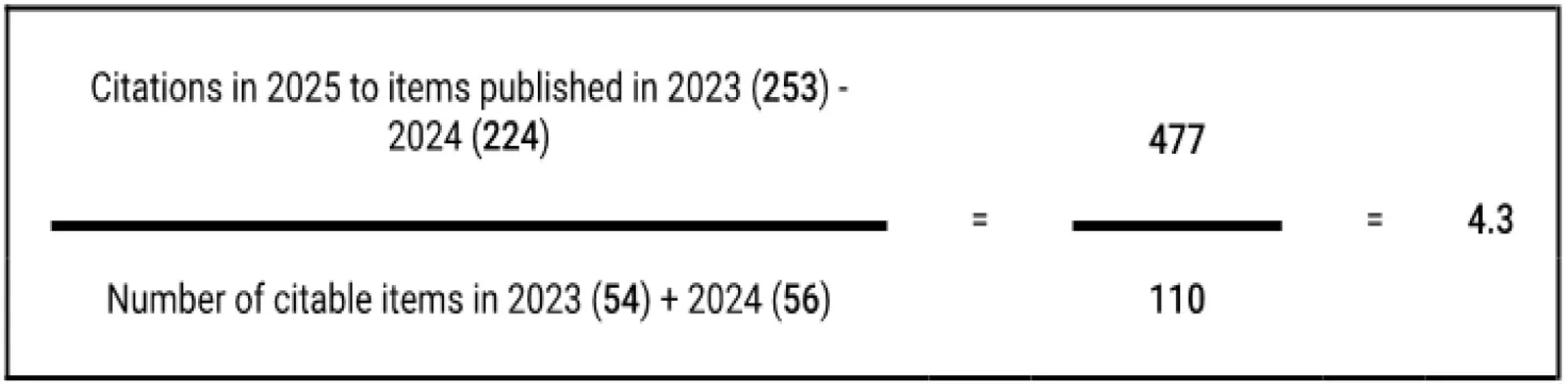
Impact factor of Int Braz J Urol in present year.

**Figure 2:**
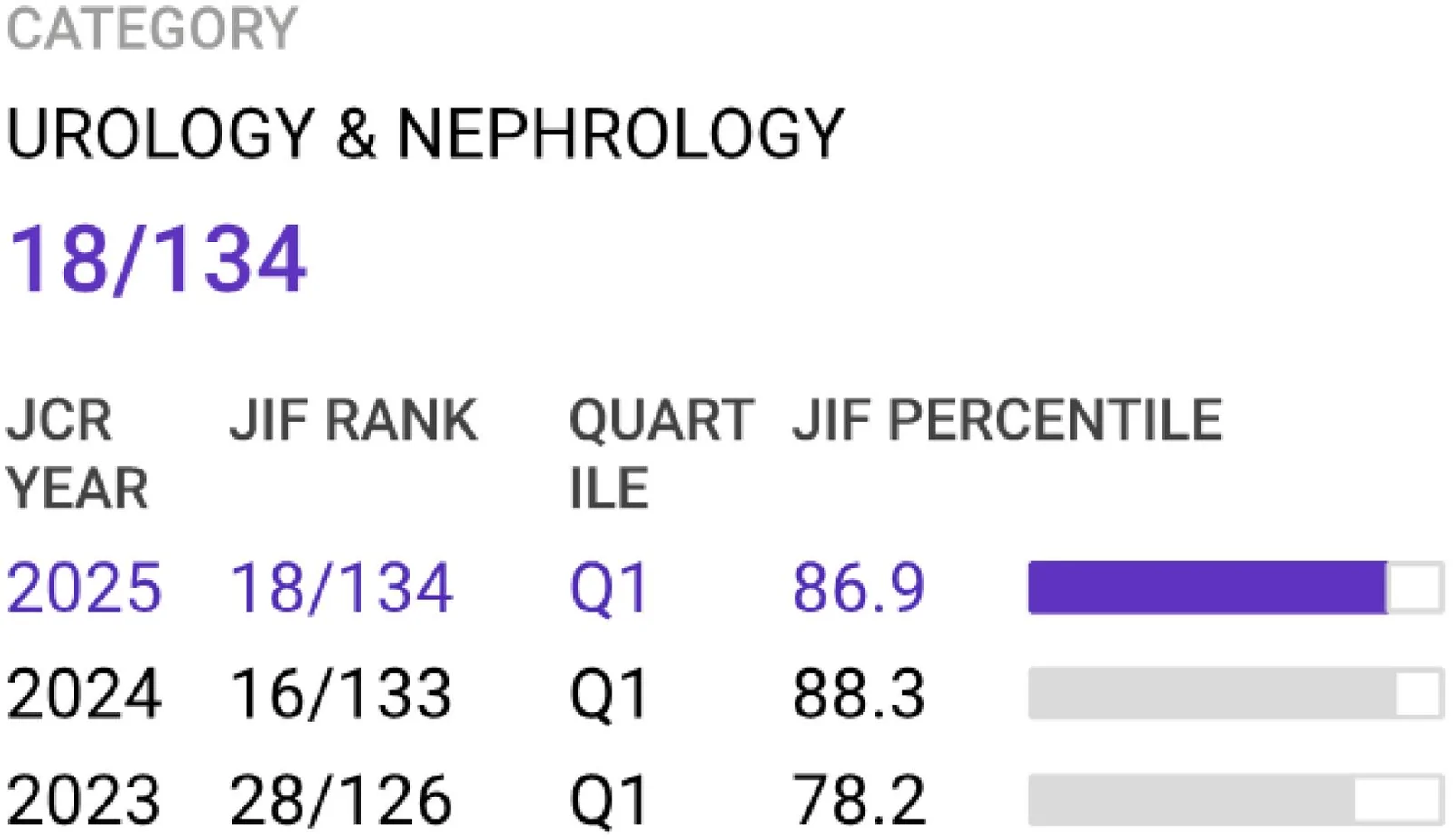
Category Ranking of The International Brazilian Journal of Urology.

The September-Octuber number of International Brazilian Journal of Urology presents original contributions with a lot of interesting papers in different fields: Prostate cancer, Robotic Surgery, Kidney cancer, Testicular cancer, Infertiliy, Erectile dysfunction and Reconstructive Urology. The papers came from many different countries such as Brazil, China, France and India, and as usual the editor's comment highlights some of them. The editor in chief would like to highlight the work about robotic surgery in kidney cancer

Dr. Pal and collegues from India, presented in page xxxx (1) a nice study about the Suture versus Sutureless Retroperitoneal Robot- assisted Partial Nephrectomy (RAPN) using VerisetTM Hemostatic Patch and concluded that VerisetTM hemostatic patch during retroperitoneal RAPN can be considered as a safe and feasible alternative to conventional cortical renorrhaphy to achieve hemostasis and the trifecta outcome. It can bring down warm ischemia time, con- sole time, and blood loss without increasing the complications and reducing the renal function. However, as the study population predominantly comprised low nephrometry score tumors, the role of this approach in moderate- and high-complexity renal masses remains to be established and warrants validation in larger studies.

Robotic surgery has revolutionized the urologic surgery, resulting in better outcomes and faster recovery (2-9). The present study is interesting and will greatly assist the urologist in treatment of kidney cancer.

The Editor-in-chief expects everyone to enjoy reading.
